# Supplementary material for: Molecular and Functional Characterization of Three General Odorant-Binding Protein 2 Genes in Cydia pomonella (Lepidoptera: Tortricidae)
Source: Int J Mol Sci. 2024 Feb 1;25(3):1746. doi: 10.3390/ijms25031746 (PMC10855334; doi:10.3390/ijms25031746)
Supplement: Supplementary file 1 [file ijms-25-01746-s001.zip › ijms-2812176-supplementary.pdf]

Table S1 Key amino acid sites of CpomGOBP2a and CpomGOBP2b with their ligands

Receptors	Ligands	CAS ID	CID	Key amino acid sites (The location number are referred to CpomGOBP2a)
CpomGOBP2a	butyl octanoate	589-75-3	11517	27V, 28T, 31F, 52F, 55F, 56W, 71I, 80L, 92M, 95Y, 109M, 113I, 130V, 133V, 134A, 137F
CpomGOBP2a	ethyl (2*E*,4*Z*)-deca-2,4-dienoate (pear easter)	3025-30-7	5281162	24M, 27V, 31F, 52F, 55F, 56W, 71I, 80L, 92M, 95Y, 109M, 113I, 130V, 133V, 134A, 137F
CpomGOBP2a	codlemone	33956-49-9	1787910	28T, 52F, 55F, 56W, 71I, 81L, 85A, 86R, 87M, 113I, 117E, 129R, 130V, 133V, 134A, 137F
CpomGOBP2a	geranylacetone	3796-70-1	1549778	24M, 27V, 28T, 31F, 52F, 55F, 56W, 71I, 75S, 80L, 92M, 95Y, 109M, 113I, 134A, 137F
CpomGOBP2b	ethyl (2*E*,4*Z*)-deca-2,4-dienoate (pear easter)	3025-30-7	5281162	33M, 36V, 37N, 40Y, 61W, 65W, 80I, 84S, 89L, 90L, 96M, 101M, 104Y, 122F, 143M, 146F

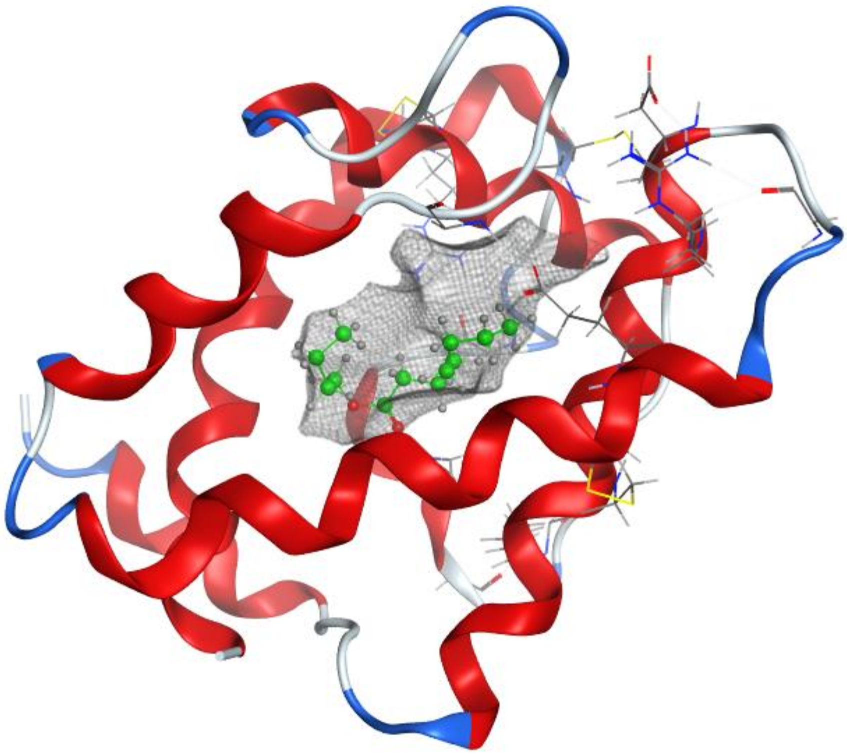

Figure S1 3D demonstration of the CpomGOBP2a binding to its butyl octanoate ligand

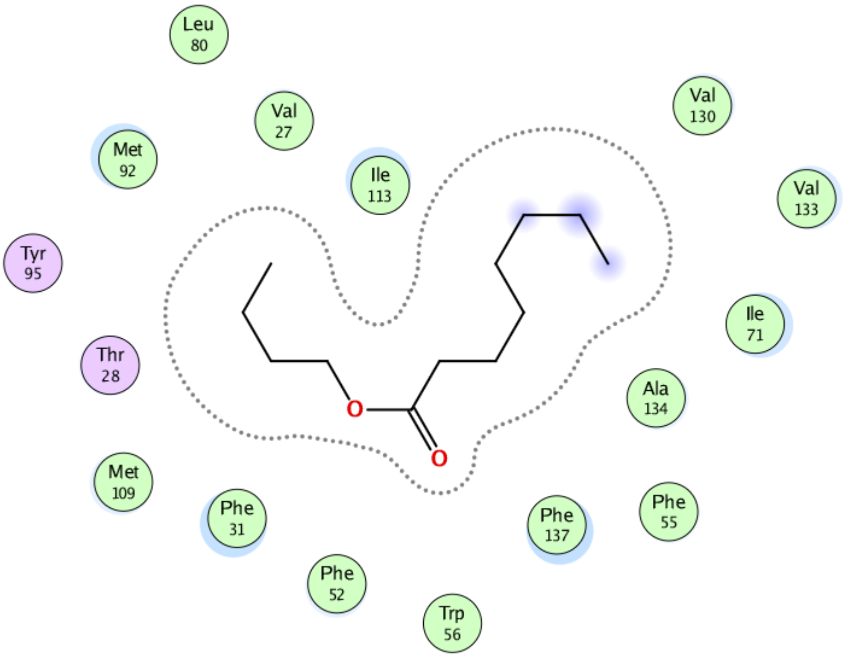

Figure S2 Key interactions at the active sites between the CpomGOBP2a and butyl octanoate

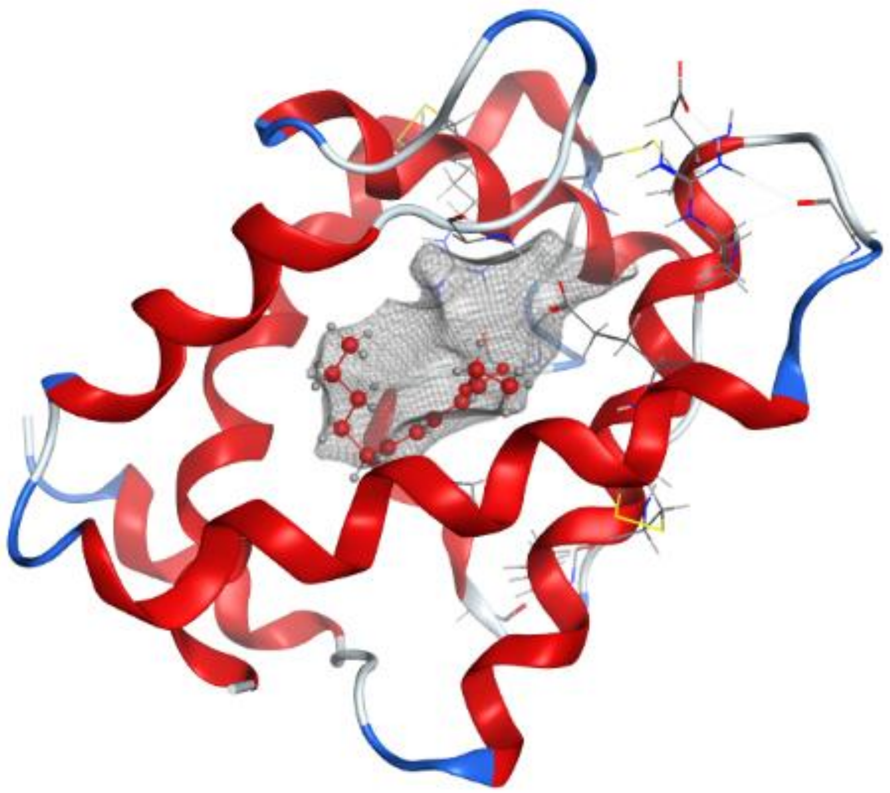

Figure S3 3D demonstration of the CpomGOBP2a binding to its ethyl (2*E*,4*Z*)-deca-2,4-dienoate (pear easter) ligand

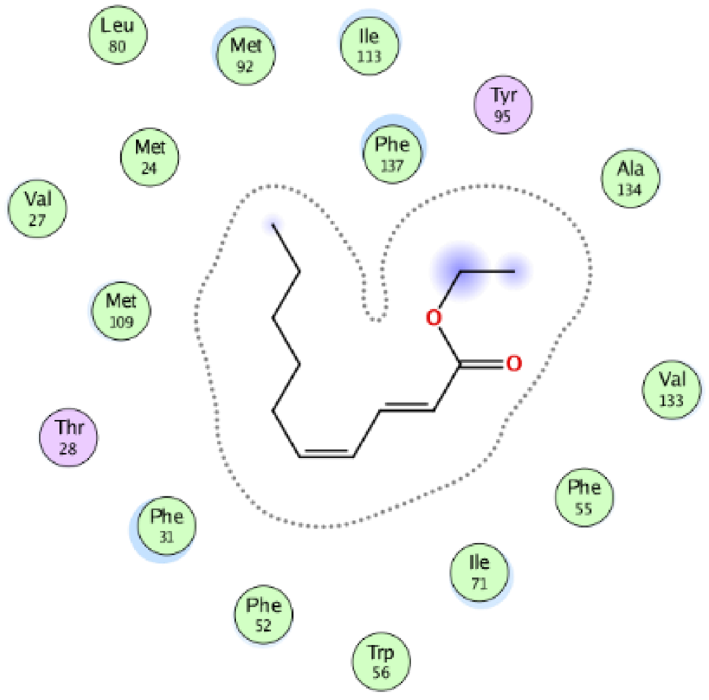

Figure S4 Key interactions at the active sites between the CpomGOBP2a and ethyl (2E,4Z)-deca-2,4-dienoate (pear easter)

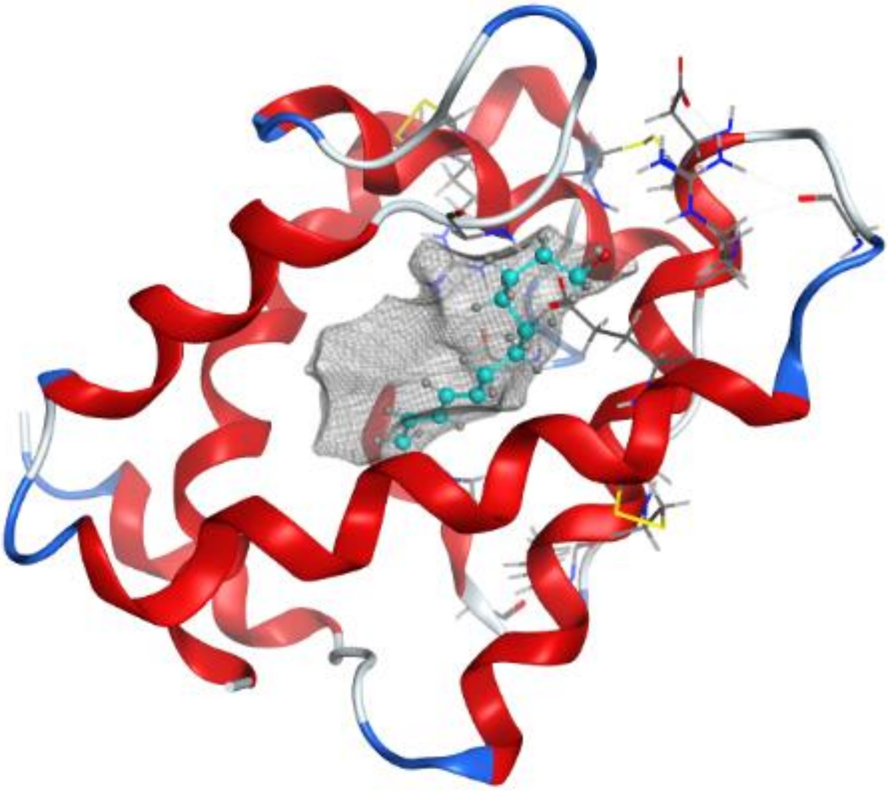

Figure S5 3D demonstration of the CpomGOBP2a binding to its codlemone ligand

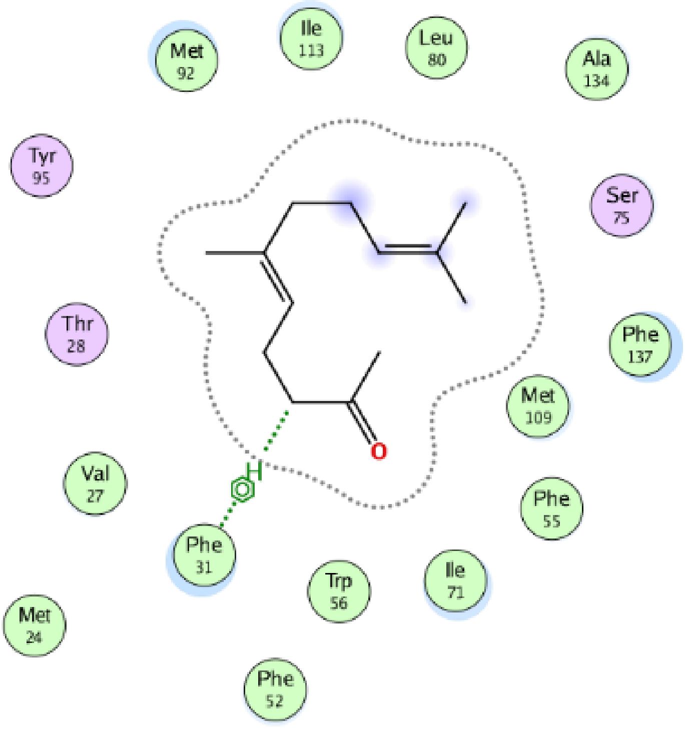

Figure S6 Key interactions at the active sites between the CpomGOBP2a and codlemone

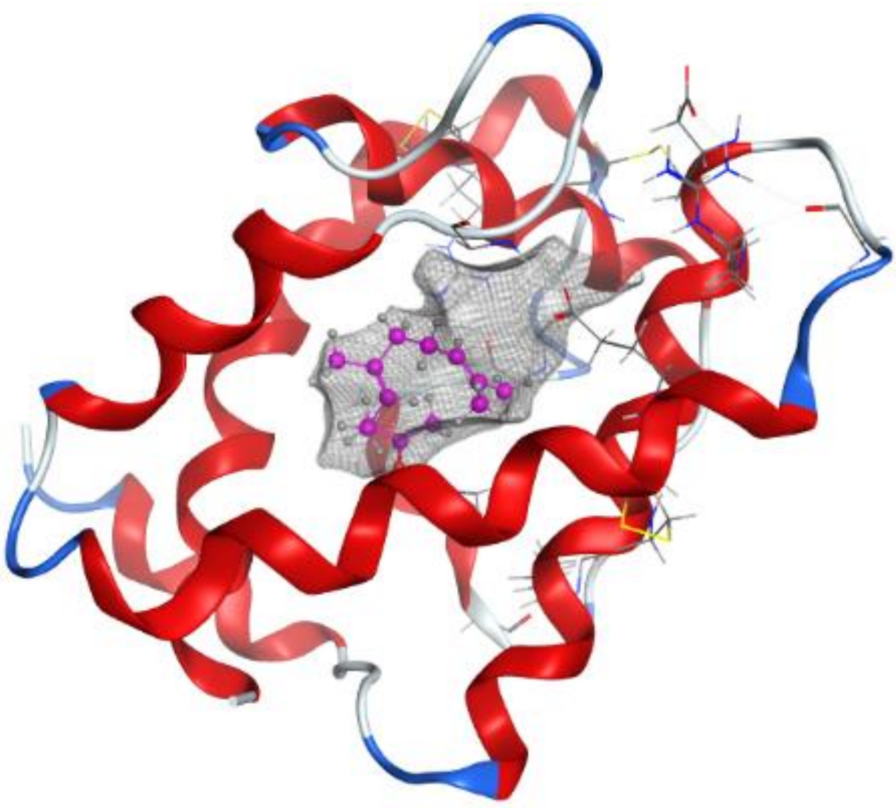

Figure S7 3D demonstration of the CpomGOBP2a binding to its geranylacetone ligand

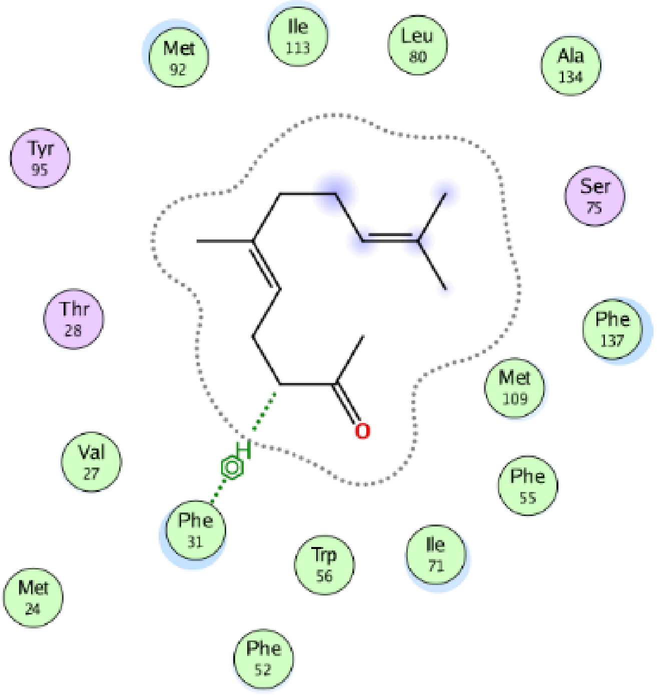

Figure S8 Key interactions at the active sites between the CpomGOBP2a and geranylacetone

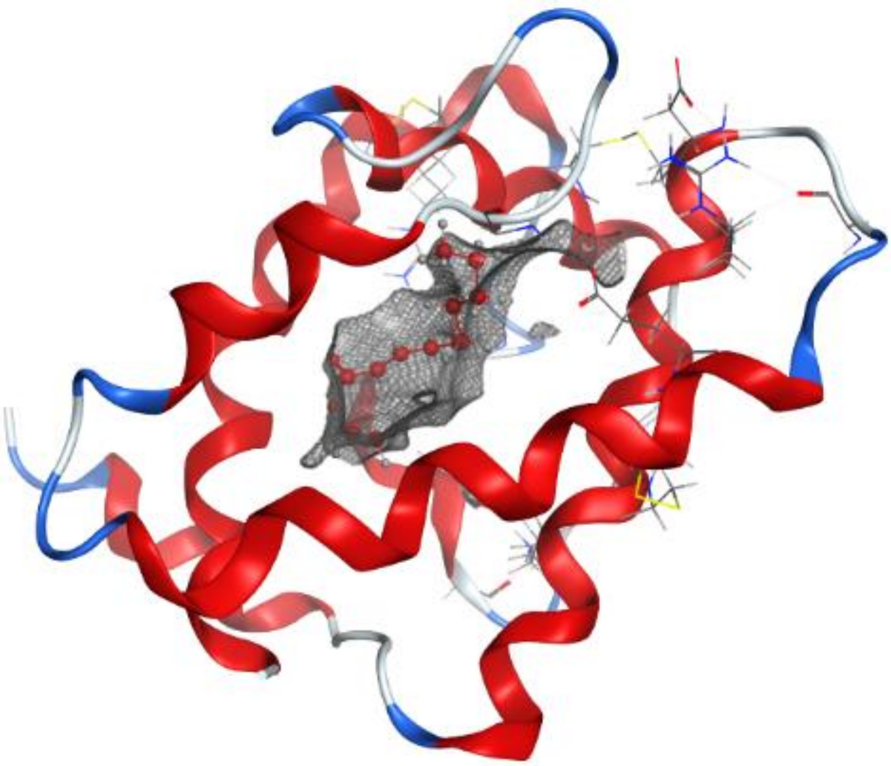

Figure S9 3D demonstration of the CpomGOBP2b binding to its ethyl (2*E*,4*Z*)-deca-2,4-dienoate (pear easter) ligand

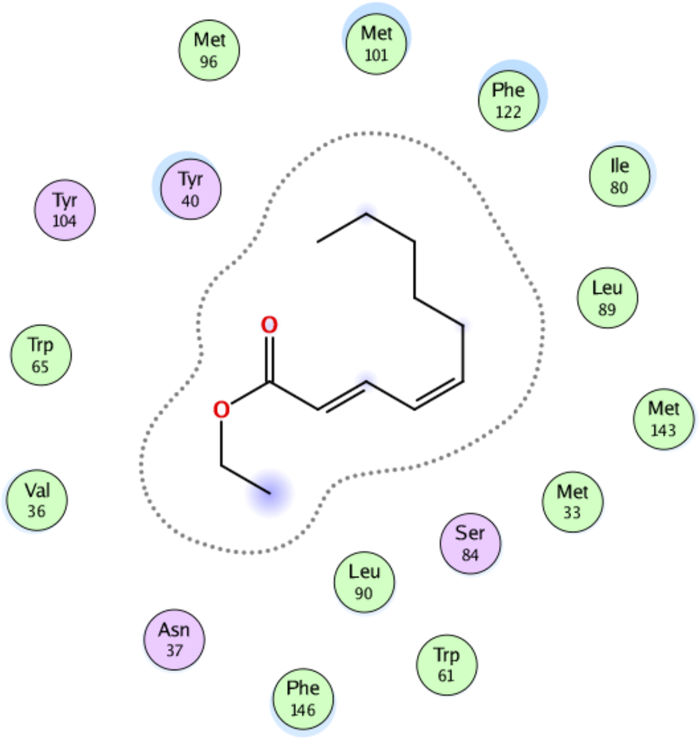

Figure S10 Key interactions at the active sites between the CpomGOBP2b and ethyl (2*E*,4*Z*)-deca-2,4-dienoate (pear easter)
